# Work–Family Guilt in Spanish Parents: Analysis of the Measurement, Antecedents and Outcomes from a Gender Perspective

**DOI:** 10.3390/ijerph18158229

**Published:** 2021-08-03

**Authors:** Olga Gómez-Ortiz, Andrea Roldán-Barrios

**Affiliations:** Department of Psychology, Facultad de Ciencias de la Educación, University of Córdoba, Avda. San Alberto Magno S/N, 14004 Córdoba, Spain; andrea.roldan.barrios@uco.es

**Keywords:** Work–Family Guilt Scale, parenthood, emotion, wellbeing, guilt, work–family conflict, personality

## Abstract

This research work had three objectives: (1) to analyze the psychometric properties of the Spanish version of the Work–Family Guilt Scale, (2) to examine its invariance according to gender, and (3) to study the relationship between work–family guilt (WFG) and the different proposed antecedent (e.g., hours spent working, social support, rumination, and personality) or consequential factors (e.g., life satisfaction), noting any gender differences. The incidental sample comprised 225 parents who were in paid work and had at least one child attending nursery school (49.1% women; age of total sample = 36.88 on average). Multiple-group and confirmatory factor analyses, correlations, multiple regression, and moderation analyses were carried out. The WFGS reflected the same factorial structure in men and women, with two main factors: work interfering with family guilt (WIFG) and family interfering with work guilt (FIWG). No gender differences were found. The discrepancy associated with perfectionism was the only variable that was found to be a predictor of FIWG. The major predictors of WIFG were brooding from rumination and the number of hours spent working. WIFG was also associated with lower life satisfaction in women. The implications of these results are discussed, stressing the need to promote work–family reconciliation policies.

## 1. Introduction

The birth of a son or daughter heralds a period of personal transition in which parents take on a new role and their identity is transformed. These new circumstances lead to changes in personal and family routines, and the parents assume new responsibilities with new experiences that can trigger a range of new emotional processes [1]. In recent times, research has focused on the emotions that arise from the difficulty of balancing the new duties involved in parenting with responsibilities at work [2,3]—in other words, when the parents experience a major work–family conflict (WFC). The emotional processes linked to WFC include a reduction in wellbeing [4,5,6]—or in life, family, and marital satisfaction [5,7]—and especially the arousal of guilt [8]. This last emotion has received much research attention in recent times [6,9,10,11]. However, it is necessary to study the guilt linked to WFC and its related factors in depth in order to better understand it and to thus be able to prevent it and its consequences.

Specific instruments have been designed to evaluate guilt related to work–family conflict, such as the Work–Family Guilt Scale [12]. Although this scale has been validated for use in different countries and has suitable psychometric properties [9,13], no specific data are available that validate the scale in the Spanish population. The first objective of this study was therefore to examine the psychometric properties of the Spanish version of the scale. Given the existing controversy over the role of gender in the occurrence and impact of guilt [14], we also adopted the following objectives: to analyze the factorial invariance of this scale according to gender, to examine possible gender differences in the dimensions of the WFGS, and to establish whether gender can moderate the effect of guilt arising from work–family conflict on general life satisfaction. Finally, we aimed to clarify the factors that give rise to this emotion, looking at both contextual and individual factors, in order to guide future interventions that can avert the guilt produced by work–family conflict and its consequences.

### 1.1. Work–Family Conflict (WFC)

One of the most accepted definitions of WFC is that given by Greenhaus and Beutell [15]. According to these authors, this conflict reduces to the perception that there is incompatibility between family and working roles, and this results in a difficulty to meet the demands of both. In this way, dedicating time to tasks in one sphere makes it more difficult to perform the tasks in the other [15], as the roles in each sphere, the support found in work or family, the characteristics of each context, and the personal involvement in them predict the perception of interference between them [16]. This conflict can be generated in either direction, with working demands interfering with family tasks (i.e., work interfering with family conflict) or vice versa (i.e., family interfering with work conflict) [17].

There are three main channels through which sources of conflict between the family and work environment arise: the time it takes to complete tasks, the pressure generated, and the feeling of overload that the roles produce [15,17]. Apart from the specific characteristics of their job and family, an individual’s personal qualities can also worsen or alleviate their perception of work–family conflict. Here, studies of personality traits have found a positive association with neuroticism and a negative link with agreeableness [18,19,20]. As regards conscientiousness, contradictory results have been found, with some studies suggesting that this trait creates greater work–family conflict [21] and others that it reduces it [18,20]. Perfectionism also seems to play a key role in fostering such conflict; in particular, maladaptive perfectionism has shown direct associations with work–family conflict [19], and it also has an indirect link by increasing parental stress and emotional exhaustion and reducing well-being [22]. On the other hand, adaptive perfectionism appears to help individuals manage WFC by increasing their self-esteem and their perception of their self-efficacy [23].

### 1.2. Work–Family Conflict and Gender

Although recent statistics have shown that European women work fewer hours than men on average [24], research evidence seem to indicate that working mothers suffer greater work–family conflict than fathers [5,25,26]. However, a recently published meta-analysis states that the effect of the difference is only slight [27]. The greatest impact on mothers arises from the couple’s unwillingness to share housework equally, which still occurs today in many countries, with the current imbalance very similar to that before the large-scale incorporation of women into the labor market [24,28]. Particularly in Spain, the main responsibility for housework and family duties still falls on women, even in cases where women work outside the home [29]. However, when both partners are in paid work, this contributes to a redistribution of these tasks, especially those of looking after a family, and this burden is to some extent shared between the partners [30].

This imbalance in the distribution of domestic tasks between partners contributes to upholding traditional gender roles, which, although they are evolving, are doing so slowly [31]. In fact, even in countries with more egalitarian views on gender equality and female employment, women are expected to be more involved in household chores and family duties [4]. These gender role attitudes further worsen work–family conflict for those who hold them, as their roles in these domains become a key part of their identity [15]. Women are therefore more vulnerable to work–family conflict, since motherhood continues to be a fundamental part of women’s lives [32,33], and the way mothers behave is still closely linked to the model of intensive maternity taken for granted in previous generations [34]. This model features intense, short-term emotional dedication aimed at ensuring the child’s wellbeing by satisfying his/her needs, with the mother’s needs relegated to second place [35]. For this reason, any attempts to combine a working role with that of wife/mother are often considered conflictive and produce a great sense of ambivalence, resulting in women experiencing feelings of guilt when their work does not allow them to be as available as they feel they should be for their family [36]. In most cases, mothers do not only perceive work as interfering in their family, as they also worry that their family duties, to which they dedicate more time than their partners [29], get in the way of their role as a worker [27].

Nor are men exempt from this conflict, in one direction or another. Gender roles are evolving, as are the beliefs held by couples, and fathers are becoming increasingly aware of the need to be involved in looking after their family and doing the housework. In fact, they often actively seek this role, and dedicate a great deal of time to their role as parents and partners [30]. Thus, the traditional male stereotype of the “breadwinner” or “head of the household” is slowly being replaced by a new model of masculinity characterized by active involvement in domestic tasks [37]. However, the roles derived from this model of masculinity are still in flux: they are neither fully defined nor assumed by all men, while the breadwinner stereotype linked to the traditional “father figure” is still dominant [33]. This puts many men in a complex dilemma, having to choose between their family responsibilities, which men consider necessary and important, and their work obligations, which men see as essential, as their work obligations are perceived as part of their main role of supporting the family. This, in turn, produces strong feelings of guilt when men do not get involved as much as they would like in either area [36].

### 1.3. Guilt Linked to Work–Family Conflict

Recently, many studies have focused on guilt linked to parenthood [6,8,9,14,36]. Since there are still few studies in this field, and those that exist are presented mainly from a managerial rather than a strictly psychological viewpoint, it is certainly worth investigating further how this feeling arises in fathers and mothers. 

Guilt is a moral emotion that stems from a person’s negative self-evaluation when they believe that their conduct, thought, or emotion diverges from their ethical code or from civic or social norms [38]. Recent neuroimaging evidence has shown that guilt is also a complex emotion because it arises from the combination of different basic emotions [39]. It is often accompanied by the perception that someone or something will be harmed, and this may impel the person to try to make amends [40]. Some approaches emphasize the social role of this emotion, as it helps in avoiding behaviors that can threaten personal relationships. However, other perspectives emphasize its connection with punishment for bad actions, leading to more than just the possible amendment of behavior and producing feelings that motivate people to avoid transgressing behaviors in the future [41]. There are two key elements in the development of this emotion: the feeling of responsibility and the perception of control. Guilt only appears, therefore, when a person feels responsible for a certain outcome, thinking that it should have been foreseen and avoided or that they should simply have acted differently [42]. This train of thought is related to the perception of control, which assumes that when the individual acted in a certain way, they were given the chance to choose between several options, and therefore had full control over their actions and the consequences they might have for others [43]. In particular, the guilt linked to work–family conflict is manifested by the need to choose between family and work duties, when both are perceived as being necessary but are also conflictive and incompatible, as they belong to different domains of the individual’s life and take up a great deal of their time or energy, which makes them difficult to reconcile [7]. When this perception of interference between activities belonging to different spheres of life is produced, any choice that leads to participating more in the activities in one sphere at the expense of the other will lead to dissatisfaction and a growing feeling of guilt [44,45]. There is currently little evidence regarding guilt linked to work–family conflict, but what there is shows that this feeling is not only very generalized, regardless of culture or country of origin [9,13,36,40,43,46], but also can be extremely diverse and manifest in many different ways, depending on the conflict that triggers it. In this context, some authors refer to guilt as having two dimensions: first, work–family guilt, produced by work interfering with family duties, and second, family–work guilt, when family responsibilities affect work [12,14].

One of the most widely used instruments for evaluating the guilt generated by the interference between family duties and obligations at work is the Work–Family Guilt Scale [12], which covers two dimensions: “work interfering with family guilt” (WIFG: four items) and “family interfering with work guilt” (FIWG: three items). This scale has been validated for use in populations from different countries, including Spain, and shows excellent cross-cultural invariance [9,13]. However, for its use in the Spanish population, the psychometric properties of the scale have not been specifically reported, and there is no Spanish version of this instrument available. Therefore, the main objective of this study was to provide psychometric data for a Spanish version of the WFGS, examining its factorial structure and invariance according to gender and its internal consistency. Previous research using the WFGS in Canada, Portugal, and cross-culturally evidenced a two-factor structure and indicated that it has very good internal consistency, with excellent convergent and discriminant validity as well as good invariance when measuring gender [9,12,13]. Although the scale has proven its factorial invariance as a function of gender and there are few gender differences in the scores of its component factors [9,13], not all previous studies have concluded the same levels of guilt linked to work–family conflict in men and women. In some research, using other evaluation procedures, higher levels of guilt linked to work–family conflict were found in mothers, especially as regards work interfering with family duties [5,6,46,47]. In others, the emphasis is slightly different, with this trend only emerging when work–family conflict is very high [10] or when the mother is required to work overtime [48]. Therefore, the second objective of this study was to check whether the average score of the component factors varies between men and women. 

### 1.4. Work–Family Guilt: Consequences and Related Factors

There are many negative effects of guilt connected to work–family conflict. At the workplace level, although FIWG encourages pro-social behavior towards colleagues, WIFG has been associated with worse performance at work, lower job satisfaction, and increased “turnover intentions”, i.e., considering quitting [11,13,14,49]. Equally damaging is the psychological impact of this emotion, as it is linked to lower satisfaction with life and family and high levels of stress, especially when family duties interfere with work, although the strength of these relationships depends on the country [13]. In all cases, not only does the existence of feelings of guilt related to work–family conflict have consequences for the parents themselves, but it also affects family dynamics, transforming parenting style and changing parental involvement in the task of looking after children [6,10,50].

Although gender differences have been a main focus of the literature on work–family conflict and its associated feelings of guilt [14], very little research has been done on the possible differences in the psychological impact of this emotion on mothers or fathers. However, the available literature points to mothers being more vulnerable to the effects of WIFG. In particular, mothers experience higher levels of stress when they prioritize work responsibilities over family duties [48,51]. Mothers are also more prone to seeing their well-being reduced as a consequence of the guilt they feel in this situation and seem to be more willing to display compensatory behavior to make amends for this feeling of guilt [6]. However, the few studies on this subject are all of a qualitative nature, or focus on work–family conflict or on general feelings of guilt rather than on the guilt associated with this conflict. In addition, we found no studies that examined the influence of gender on the guilt generated by family interference in work. Another objective of this research was therefore to examine what influence the two dimensions of the WFGS have on life satisfaction and the moderating role of gender. 

The personal impact of the feelings arising from a perceived conflict between work and family can be very severe, and it is therefore useful to examine the factors that cause it in order to help prevent it.

Among the factors linked to the development of WFG, the most important relates to the time available to dedicate to the two spheres. Here, the hours spent at work can be an accurate predictor of the guilt generated by the interference of work in the family [13], and this seems to especially affect women [10]. A link has also been found between the type of working day and WIFG, with this feeling being more prevalent in parents who work full-time compared with those who work part-time [13].

Social support also affects the feelings expressed by parents, although the effects of this variable and gender on WFG are complex and depend on the source, domain, and type of support, as well as the direction of the WFG studied [14]. Aycan and Eskin [5] found that for women, but not for men, the greater the emotional support given by the partner and the boss/manager, the lower the conflict generated by the interference of work in the family, and as a consequence, the lower the work-induced guilt. A study conducted by Uysal Irak et al. [52] in a female population found the same trend. Liu et al. [53] also related high task fairness, which depends on the way that a boss or supervisor manages a work team, with a lower sense of guilt regarding the family and fewer complaints from family members.

Apart from the contextual circumstances, individual factors also affect the perception of work–family conflict and the associated feelings of guilt. The limited literature available emphasizes the roles of the five major personality traits, as well as other factors, such as perfectionism [18,19,20,21,23]. However, these five main personality traits have not been directly linked to the development of WFG, although perfectionism has been associated both with the development of general guilt [54] and with the family guilt and negative behavior displayed by parents when they experience a major conflict between these two spheres of life [55]. In addition, certain cognitive processes, such as rumination, have also been linked to general guilt [56]. Rumination can be defined as a series of continuous thoughts revolving around a common theme and recurring without direct environmental causation [57]. Although some authors assume that this is a maladaptive emotional regulation strategy, since it is linked with several emotional problems [58,59], others claim it serves a functional purpose as a response to the gap between the stated objectives and the results achieved, which drives a person to achieve the former [57]. Considering both perspectives, the existence of two forms of rumination has been proposed: adaptive rumination, or “reflective pondering”, and maladaptive rumination, which operates through brooding and self-reproach [60].

Although previous literature has examined the relationship between different factors and the development of general guilt or guilt linked to work–family conflict, we found no studies that examined how the predictive capacities of different factors contribute to the evolution of this emotion or whether there are any gender differences. The final objective of this research was therefore to study the possible influence of the number of hours worked, social support, the five main personality traits, perfectionism, and rumination on the two dimensions of work–family guilt, in order to clarify which of these factors is more determinant of this phenomenon in men and women. These results will help us design interventions aimed at preventing WFG, reconciling both the spheres of life and ultimately promoting the wellbeing of dual-earner couples.

Taking into account the specific aims of this study, the following hypotheses were formulated:

**Hypothesis** **1** **(H1).** *We hypothesized that the WFGS would display suitable psychometric properties, showing the same factorial structure in both men and women [9,12,13]*;

**Hypothesis** **2** **(H2).** *We expected to find no gender differences in either the WIFG or the FIWG dimension scores, judging from the results of previous studies that have used the WFGS for this purpose [9,13]*;

**Hypothesis** **3** **(H3).** *We expected to find a negative relationship between WIFG and life satisfaction [8], with lower levels of life satisfaction in mothers experiencing WIFG [6]*;

**Hypothesis** **4a** **(H4a).** *We hypothesized that to predict WIFG, the most important contextual factor would be working hours, as the time devoted to work is one of the major sources of work–family conflict [15,17], and consequently, of WIFG [13]—a factor would be especially important in mothers [10], as it makes them feel they are unavailable at home, which goes against the gender role beliefs and attitudes that still persist [35,36];*

**Hypothesis** **4b** **(H4b).** *We hypothesized that the perceived gap between standards set and results achieved would be the factor contributing the most to the feeling of guilt linked to work–family conflict [19,21], producing a greater perception that family duties interfere with work, since the latter involves more clearly defined obligations and expectations [22]*.

## 2. Materials and Methods

### 2.1. Participants

The reference population for this study was Spanish fathers and mothers of schoolchildren in the first and second cycles of nursery school education (aged 0 to 5 years) who were in paid work. An incidental sampling approach was carried out to select the sample. In this sense, the sample was chosen for its proximity and accessibility since the permission of the schools was required to access the sample. A total of twelve nursery schools from Córdoba and Badajoz (south of Spain) made up the sample. The final sample was made up of 225 fathers and mothers. The ages of the participants ranged between 23 and 52 years (M = 36.88; SD = 4.96). The participants worked between 3 and 14 h a day (M = 7.67; SD = 1.75), and 41.8% had one child, 53.8% had two children, and 4.4% had three children. Regarding their living circumstances, 47.8% of the sample lived in small towns (fewer than 10,000 inhabitants), 50.4% in the city (provincial capital: 325,701 inhabitants), and 1.8% in houses in the countryside. As they could be important for the interpretation of results, some data are offered that take into account gender differences. The men (50.9% of the total sample) were aged between 25 and 57 years (M = 38.12, SD = 4.94) and worked 8.27 h each day on average (SD = 1.56). They had 1.65 children on average and 96.5% lived with their partner. The women’s ages were between 23 and 48 years (M = 35.60, SD = 4.69), and they worked 7.02 h a day on average (SD = 1.72). They had 1.60 children on average, and 93.6% lived with their partner. Among the limitations of this research are the sample size and its non-representativeness of the population.

### 2.2. Instruments

#### 2.2.1. The Work–Family Guilt Scale (WFGS)

This scale, designed originally by McElwain [12], allowed us to identify the presence of guilt produced by the intersection between work and family. The scale consists of 7 items, organized into two factors: work interference with family guilt (WIFG: 4 items, e.g., I regret not being around for my family as much as I would like to) and family interference with work guilt (FIWG: 3 items, e.g., I feel bad because I frequently have to take time away from work to deal with issues happening at home). Following the standards of the most recent validation [9], the items are rated on a 1–7 Likert scale (1 = strongly disagree, 7 = strongly agree). The internal consistency of the scale and its factors is excellent, showing values >0.80 in most of the samples for which this instrument has been validated [9,13].

#### 2.2.2. The Short Form of the Almost Perfect Scale–Revised (SAPS)

This instrument [61] consists of an 8-item scale with 7 Likert-type response options (1 = strongly disagree, 7 = strongly agree), and it covers two dimensions: standards (e.g., I set very high standards for myself), which assesses expectations of personal performance, and discrepancy (e.g., My performance rarely measures up to my standards), which refers to self-critical perfectionism. In this study, the internal consistency of both the general scale (α = 0.76) and the subscales (αstandards = 0.77; αdiscrepancy = 0.79) was suitable. The AFC results were suitable: S-B χ_2_ = 81.05 (19); *p* = 0.000; NNFI = 0.90; CFI = 0.91; RMSEA = 0.12; 90% confidence interval for RMSEA = 0.09–0.15.

#### 2.2.3. The Satisfaction with Life Scale (SWLS)

This scale was designed by Diener et al. [62] and validated for use in the Spanish population by Moyano-Díaz et al. [63]. It consists of five items (e.g., The conditions of my life are excellent) with a Likert-type response scale (1 = strongly disagree, 5 = strongly agree). The internal consistency shown in the present study was satisfactory (α = 0.82).

#### 2.2.4. The Ruminative Responses Scale (RRS)

This scale was designed by Nolen-Hoeksema and Morrow [64] and adapted for the Spanish Language by Hervás [65]. It consists of ten Likert-type items (1 = hardly ever, 4 = nearly always) organized into two factors: reflection (reflective pondering, e.g., Write down what you are thinking and analyze it) and brooding (tendency to self-reproach, e.g., Thinking “Why do I have problems other people don’t have?”). In the present study, this scale offered a good internal consistency index (α = 0.83), as did its dimensions (αreflection = 0.78; αbrooding = 0.78).

#### 2.2.5. The Multidimensional Scale of Perceived Social Support (MSPSS)

This instrument was designed by Zimet et al. [66] and adapted for the Spanish Language by Landeta and Calvete [67]. It evaluates the levels of perceived social support through 12 items with 7 Likert-type response options (1 = strongly disagree, 7 = strongly agree). It consists of three factors, each referring to a person/group who give support: a significant other (e.g., There is a special person who is around when I am in need), family (e.g., My family really tries to help me), and friends (e.g., I have friends with whom I can share my joys and sorrows). The scale presents good internal consistency (α = 0.90), as do the 3 subscales (αsignificant other = 0.89, αfamily = 0.88, αfriends = 0.91).

The number of hours spent working each day was evaluated by directly asking the participants.

### 2.3. Procedure

To start with, the WFGS was translated into Spanish via “parallel back-translation” [68]. As regards data collection, permission was requested from the schools to access the children’s parents as possible participants. The schools gave the questionnaires and consent forms to the families to complete at home and explained that their participation was voluntary, anonymous, and confidential. The instructions specified that if the parents are together and both decide to fill out the questionnaire, they should do so without sharing any information with their partner. The existence of couple relationships among the participants was not considered, so their responses were independent from each other.

### 2.4. Data Analysis

Descriptive statistics were explored as a preliminary analysis. To establish the validity based on the internal structure of the questionnaire and to determine if the original factorial structure could be replicated, we also conducted a confirmatory factor analysis (CFA). Given the ordinal nature of the questionnaire’s variables, the maximum likelihood estimation method with robust correction was used [69]. The model adjustment was evaluated using the comparative fit index (CFI), the non-normed fit index (NNFI) (≥0.95), the standardized root-mean-square residual (SRMR) and the root-mean-square error of approximation (RMSEA) (≤0.08) [70]. A multiple-group analysis was then conducted to generalize the model across the gender groups. This approach compares a set of increasingly restrictive models. In this case, four models were compared: model 1, in which the same factorial structure was applied to all the groups (configural invariance); model 2, in which the covariances were restricted and kept equal between the groups; model 3, in which the factor loadings were restricted and kept equal between the groups (metric invariance); and model 4, in which both the factor loadings and the covariances were restricted and kept equal between the groups (residual invariance) [71]. The invariance was assessed in light of the results of the chi-square differentiation test (Δχ^2^, and nonsignificant changes were taken as indicative of invariance between groups [72]. Furthermore, we assessed the differences between the other fit indexes (NNFI, CFI, RMSEA, and SRMR), wherein changes ≤0.01 were taken to indicate the presence of invariance [73]. The CFA and multiple-group analysis were conducted using the EQS 6.2 program [74]. The reliability of the scale and subscales was calculated using Cronbach’s alpha (α > 0.70).

Independent *t*-tests were carried out to determine possible gender differences among the variables. To examine the relationship between the two forms of guilt linked to work–family conflict and the other variables, we calculated the correlations between them.

To test the moderation effect of gender on the relationship between WFG and life satisfaction, the PROCESS macro for SPSS was used [75]. The significance of the conditional direct effects was estimated through bias-corrected bootstrap confidence intervals (CI) derived from 5000 bootstrap resamples. The Johnson–Neyman technique was used to determine from these values for gender whether the association between work–family guilt and life satisfaction was significant. The significance level adopted for all analyses was 0.05.

Finally, to determine the extent to which the contextual and individual variables could act as antecedents of work–family guilt, a multiple linear regression analysis was performed, using the successive step method. The WIFG and FIWG variables were used as the criterion variable, and the hours spent at work, the different kinds of social support, and the questionnaire scores for rumination, SAP-S, and Mini-IPIP were used as predictor variables. In any case, the cross-sectional nature of the study did not allow causal relationships to be established, which means that all influencing relationships should be interpreted with caution. The regression analysis was carried out by specifically selecting men and women. The independent t-test, correlation, and regression analyses were performed using SPSS 23.0 (IBM Corp., Chicago, IL, USA).

## 3. Results

### 3.1. Descriptive Analyses

Table 1 shows the means, typical deviations, and indexes of skewness and kurtosis for each item of the WFGS. The highest mean was 4.09 (I regret not being around for my family as much as I would like to) and the lowest was 1.79 (I feel bad because I frequently have to take time away from work to deal with issues happening at home).

### 3.2. Multiple-Group, Confirmatory Factorial Analyses, and Gender Differences

**Figure 1:**
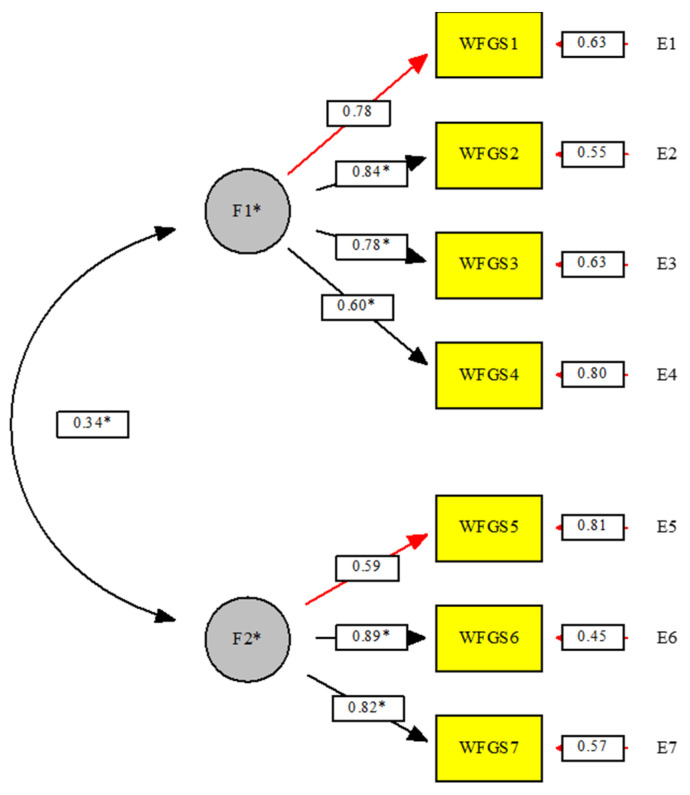
CFA standardized coefficients of the items in the WFGS. Note: * = *p* ≤ 0.05.

The results of the CFA verify the original factorial structure of the two factors (WIFG and FIWG), showing the following fit indexes: χ^2^ S-B = 22.59 (13); *p* = 0.04; NNFI = 0.98; CFI = 0.98; SRMR = 0.06; RMSEA = 0.07. All the factor loadings were significant and high (0.60 ≤ λ s ≤ 0.89) (see Figure 1). The standardized factor loadings (*R*^2^) for each item ranged between 0.34 and 0.79 and can be seen in Table 1. 

In the multiple-group analysis, four progressively more restricted models were compared. The chi-squared differences were not significant between models 1 and 2 (0.33; *p* = 0.86), models 1 and 3 (5.78, *p* = 0.80), or models 1 and 4 (5.96, *p* = 0.87). In addition, the changes in CFI, NNFI, RMSEA, and SRMR were minimal in all the comparisons (see Table 2).

No gender differences were found in either the WIFG scale (t(206) = −0.27; *p* = 0.78; mean women = 3.92; mean men = 3.85) or in the FIWG (t(214) = 1.16; *p* = 0.24; mean women = 2.07; mean men = 2.27). The scale showed an acceptable internal consistency (α = 0.78), as did the factors (αWIFG = 0.80; αFIWG = 0.69).

### 3.3. Relationship between Work–Family Guilt, Life Satisfaction, Social Support, Perfectionism, Ruminative Responses, and the Big Five Personality Traits

The correlation analysis (see Table 3) showed the following significant relationships: FIWG was directly related to perfectionism and working hours, while WIFG was positively related to the brooding dimension of rumination and working hours and negatively related to openness to experience. None of the other relationships were significant.

**Figure 2:**
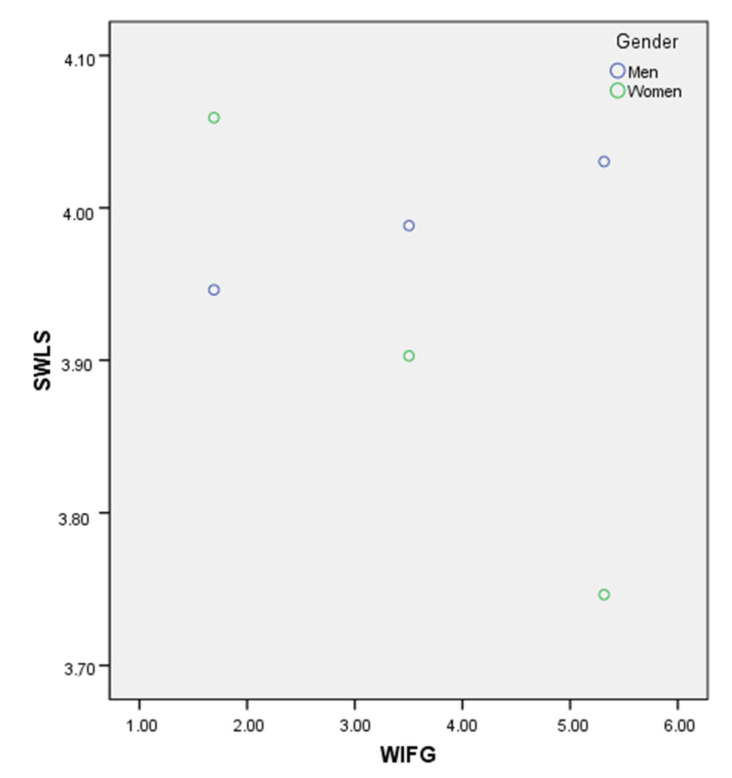
Johnson–Neyman plot of the moderating effect of gender on the relationship between satisfaction with life (SWLS) and work interference with family guilt (WIFG).

In the moderation analysis, although no direct relation was found between gender (β = 0.35, SE = 0.24, *p* > 0.05) or WIFG (β = 0.12, SE = 0.09, *p* > 0.05) and life satisfaction, it was clear that the effect of the former variable on life satisfaction was moderated by gender (β = −0.11, SE = 0.05, *p* < 0.05). Assessment via the Johnson–Neyman technique showed that the positive association between WIFG and life satisfaction was only significant for women, who constituted 49.1% of the participants (see Figure 2), and no significant relationships were found between FIWG and life satisfaction, either directly (β = 0.15, SE = 0.12, *p* > 0.05) or as moderated by gender (β = −0.09, SE = 0.08, *p* > 0.05).

The results of the regression analyses are also shown schematically in Table 4. The model for predicting WIFG in women was significant (*F*(78) = 12.07; *p* ≤ 0.001), with self-reproach and hours spent at work selected as the predictive variables, which accounted for 22.1% of the variance in the WIFG. The model was also significant when applied to men (*F*(84) = 6.95; *p* ≤ 0.001), and it established the same two predictive variables as in women, with the addition of openness to experience. This model accounted for 17.5% of the WFG variance.

Meanwhile, the FIWG prediction model was significant in both women (*F*(81) = 7.73; *p* ≤ 0.01) and men (*F*(86) = 9.19; *p* ≤ 0.01). In all cases, the discrepancy related to perfectionism was selected as the predictor variable. The models accounted for 7.7% and 8.7% of the variance in the FIWG for women and men, respectively.

## 4. Discussion

The main aim of this study was to verify the psychometric properties of the Spanish version of the Work–Family Guilt Scale [12]. These properties were good, with the scale particularly showing suitable internal validity, with a structure made up of two factors: WIFG and FIWG. As we had hypothesized, this pattern was consistently invariant across gender groups, in line with previous studies [9,13]. Both factors showed adequate internal consistency. 

No gender differences were found in either of the two dimensions of the WFGS, which thus confirms our second hypothesis. These results coincide with those of previous studies that used the same scale [9,14] but not with those that used different assessment instruments or procedures [5,6,46,47]. This highlights the need to standardize the measuring instruments used for evaluating guilt linked to work–family conflict. This would allow us to draw more robust conclusions about the existence of this emotion in fathers and mothers and the possible differences between them. The results obtained, when interpreted in light of the existing literature, show that men and women manifest similar levels of guilt generated by the interference of work in the family, or vice versa. These findings also correspond with men’s increasing involvement in housework and family responsibilities [30,37], which makes them just as vulnerable as women to experiencing a conflict between work and family life [27], and therefore, vulnerable to developing feelings of guilt linked to this conflict. Furthermore, it is important to consider not only the constraints on time at home but also those at work because both are important sources of work–family conflict [16,17], and hence, guilt [8]. In this sense, it is not surprising to find no gender differences because although women dedicate more time to domestic and family tasks, men’s average paid work hours are still higher than those of women, as can be found via international comparisons [24] and also in the results of this study. Nevertheless, these results could be moderated by the influence of certain variables, such as the level of work–family conflict or, particularly, the circumstances in the workplace or in the family context, as other studies have shown [10,48].

On the other hand, the results of this study did reveal gender differences in the impact of WIFG on life satisfaction. In particular, it was found that the relationship between WIFG and life satisfaction was only significant for women. However, no significant relationship was found between FIWG and life satisfaction, nor was this relationship moderated by gender. Furthermore, the results of the correlation analysis did not reveal any significant relationship between life satisfaction and either of the two dimensions of the WFGS scale, so our third hypothesis was only partially confirmed. These results are not fully consistent with the existing literature, in which WIFG is inversely related to life satisfaction, although the effect size of this relationship seems to depend on the country (which in Spain is low) [8]. Further research is required in order to clarify the relationship between these two constructs, in which not only culture but also gender seem to play a key role. From this viewpoint, other studies report more profound consequences for mothers when they prioritize work responsibilities over family duties [48,51], as well as more guilt related to handling this balance [6]. Our results are extremely interesting from the point of view of gender differences because they show that although men and women may display similar levels of guilt linked to work–family conflict, the latter are affected more, especially when the feeling of guilt arises from the perception that work is interfering with family obligations. One explanation for this could be found in the gender roles and beliefs that still prevail in our society, since holding down a paid job makes it impossible for women to fulfil the role of mother and/or wife with all the commitment and availability that is traditionally expected [35]. This ideal of motherhood is still latent in today’s society [4,34] and means women experience significant feelings of guilt when they fail to meet the implicit conventions [36], with the assumption that their behavior could in some way be harmful to their children or to the family as a whole [40] and that it is all the result of a voluntary choice [43]. The feeling of remorse produced by this failure to adhere to gender role standards has a significant personal impact [41]. However, in addition, recent qualitative studies suggest that as a result of this feeling of guilt, mothers are even more prone than fathers to displaying compensatory behavior to alleviate this guilt. One result of this is that women sacrifice their leisure time or personal time and dedicate more time to their children, thus adjusting to prevailing gender norms [6], which could have a knock-on effect on their life satisfaction. Further studies need to be conducted in this field to determine how guilt linked to work–family conflict ends up compromising mothers’ life satisfaction. The existing studies suggest that, despite the fact that gender roles and beliefs are evolving, fathers continue to take greater responsibility for economically supporting the family than looking after it [24,36,46], which could account for the lesser impact of this kind of guilt in this group. Further research, however, is required to draw more robust conclusions.

The two predictor variables that best predicted WIFG were brooding resulting from rumination and hours dedicated to work. In men, openness to experience was the most noteworthy predictor variable among the factors of personality.

Although, to the best of our knowledge, this is the first time that the relationship between WIFG and brooding has been reported, it is not an unusual finding if we take into account the relationship between rumination and general feelings of guilt [56]. As we found in this study, self-reproach could be one of the main causes of WIFG. If these thoughts are not regulated, they could well lead to an imbalance, in line with findings from previous research [59,60,64,65]. However, they could also represent a moral tool [40] for inducing compensatory behavior in the parents, which modulates their presence and availability in the home [6,50]. The utility of this emotional regulation strategy should thus be evaluated to decide whether the guilt linked to work–family conflict could have the adaptive function that Martin and Tesser [57] attributed to it, as well as examining any possible gender differences in this variable [64].

The only contextual variable linked to WIFG was the number of hours spent at work, which partly confirms H4a, as this relationship was found in both men and women. This result is consistent with those from the previous literature that identified the time factor as not only one of the main sources of work–family conflict [15,17] but also as an important predictor of WIFG [13], especially in women [10]. The fact that in our study it is also a powerful predictor of WIFG in men could imply two things: first, its robustness as a predictor of this emotion, and second, taking into account previous results that found that both working parents seem to share this feeling, albeit with different implications, and that it is triggered by very similar factors. It may be that differences in this aspect depend more on a person’s orientation and adherence to gender roles or culture than simply on gender itself [45]. This would reinforce the idea that in countries such as Spain, where great importance is attached to the family and a growing number of individuals are showing a preference for developing a symmetrical or egalitarian family model (in which both partners work outside the home and share the housework and childcare) [76], men and women are equally sensitive to the interference of work. Further studies are needed to confirm this theory.

Although previous studies have highlighted social support as a key factor in the experience of work–family conflict and its derived feelings of guilt [5,6,7,8,9,10,11,12,13,14,15,16,17,18,19,20,21,22,23,24,25,26,27,28,29,30,31,32,33,34,35,36,37,38,39,40,41,42,43,44,45,46,47,48,49,50,51,52], this was not found in our study. It could be that when social support competes with other variables of a different nature, its ability to predict the guilt linked to work–family conflict decreases, thus relegating it to a secondary predictor of this emotion. Another possible reason is that the type of support evaluated on this scale may not be so relevant in explaining WFG [14]. These questions need to be clarified in future research.

The only personality trait found to be linked to WIFG was openness to experience, but this was only in men, and it was inversed. Our findings seem to indicate that men who are more open to experience are less likely to experience WIFG. To the best of our knowledge, no previous studies have linked openness to experience with guilt linked to work–family conflict. Perhaps, the interest in diverse cultural, artistic, or literary matters inherent in this trait leads to greater involvement in activities that distract from this feeling and, as such, regulate it [77]. However, if the individual does not have enough time to get involved in these activities because their domestic responsibilities do not permit it [28,29], or if they dedicate all their free time to their children to make up for work-related absences [6], this trait may not provide effective protection. This situation may underlie the fact that “openness to experience” does not feature in women as a protective element of WIFG, although this needs to be verified empirically.

The only variable to emerge as a predictor of FIWG in both men and women was the discrepancy related to perfectionism, thus confirming Hypothesis 4b. Although there has been little research into the relationship between perfectionism and guilt linked to work–family conflict, the maladaptive version of this feature has been found to worsen work–family conflict [19], to generate guilt and stress in the family [55], and to lead to cynicism and exhaustion at work [22]. Our results agree with this, showing that individuals who perceive important discrepancies between their life goals and their results are at greater risk of feeling guilty as a consequence of FIWG. As we hypothesized previously, it is quite likely that a relationship exists between discrepancy and FIWG simply because the workplace offers better-defined goals, with clearer targets set, therefore inducing the possibility of perceiving discrepancies in reaching them [22]. When these discrepancies at work are felt to have resulted from family interference, FIWG arises.

The results of this research have different practical implications. On the one hand, they highlight the need to improve reconciliation policies in Spain, especially those concerning reducing the amount of time spent at work to promote greater involvement at the family level and thus mitigate WIFG. From this viewpoint, a reduction should be considered in working hours for parents with children in education, or other measures such as flexible schedules, goal-orientated work objectives, working remotely, or compressed work weeks [78,79]. Furthermore, this study shows that work–family conflict and the feelings that emerge from it depend not only on the conditions of both contexts, which are beyond the individual’s control, but also on the individuals themselves. This provides an optimistic vision for handling this conflict, in that it is not only up to the state or private companies to endorse conciliation policies (and who may adhere to these policies to a greater or lesser extent), but it is also up to the individual. In this regard, any psychological intervention, in light of the results obtained, should focus on three essential cornerstones: (1) promoting effective strategies for emotional regulation to prevent feelings of self-reproach when parents are simply unable to spend more time with the family; (2) regulating perfectionism by detecting discrepancies and encouraging a more realistic approach to working standards adjusted to the situation of being a parent; (3) encouraging personal time management in which self-care has a place, especially in mothers, who are prone to sacrificing this time in order to look after their children [6,35].

Among the limitations of this research are the sample size and its lack of representativeness of the population. Likewise, the cross-sectional nature of the study does not allow causal relationships to be established, which means that all influencing relationships should be interpreted with caution. In future research, longitudinal and cross-cultural studies would be needed to confirm these relationships and others arising from this discussion.

## 5. Conclusions

The results of this research verify the validity and reliability of the Spanish version of the WFGS. They also confirm the absence of gender differences regarding WFG—although differences do exist in the impact of this emotion—as well as establishing some of the factors related to this guilt and which could thus contribute to its prevention. Regarding these factors, the number of hours spent at work and the engagement in brooding were the most important predictors of WIFG, while the discrepancy related to perfectionism was the only variable related to FIWG. In order to mitigate these feelings, and thus favor parents’ and children’s wellbeing, families need to feel more supported by the State, with more measures to better reconcile family and work life.

## Figures and Tables

**Table 1:** English and Spanish items of WFGS, descriptive statistics, and standardized factor loadings of AFC (*R*^2^).

	M	SD	S	K	*R* ^2^
1. I regret not being around for my family as much as I would like to (Me arrepiento de no estar cerca de mi familia tanto como me gustaría)	4.09	2.36	−0.13	−1.54	0.60
2. I feel guilty for not being able to take care of my child(ren) as well as I would like to (Me siento culpable por no poder cuidar a mi(s) hijo/a(s) tan bien como me gustaría)	3.46	2.15	0.25	−1.39	0.69
3. I feel bad because I frequently have to take time away from my family to deal with issues happening at work (Me siento mal porque con frecuencia tengo que dejar a mi familia para ocuparme de asuntos profesionales o laborales)	4.03	2.2	−0.07	−1.43	0.60
4. I feel guilty for not showing as much interest in my spouse/partner as I wish (Me siento culpable por no mostrarle a mi pareja tanto interés como me gustaría)	3.92	2.12	−0.08	−1.39	0.36
5. I am worried about the quality of my work because I often put my family before my job (Me preocupa la calidad de mi trabajo porque a menudo antepongo mi familia al trabajo)	2.97	1.95	0.53	−1.02	0.34
6. I regret missing work due to family responsibilities (Me arrepiento de haber faltado al trabajo debido a mis responsabilidades familiares)	1.82	1.41	1.71	2.13	0.79
7. I feel bad because I frequently have to take time away from work to deal with issues happening at home (Me siento mal porque tengo que ausentarme frecuentemente del trabajo para lidiar con los problemas que suceden en casa)	1.79	1.37	1.93	3.32	0.68

Note. M = mean; SD = standard deviation; S = skewness; K = kurtosis; *R*^2^ = standarized factor loading.

**Table 2:** Multiple-group analysis of WFGS: configural, metric, and residual invariance.

Models	S-B χ^2^	*df*	S-B χ^2^/*df*	*p*	NNFI	CFI	RMSEA	SRMR	∆ S-B χ^2^	*p*	∆*df*	∆NNFI	∆CFI	∆RMSEA	∆SRMR
Model 1	35.70	26	1.37	0.09	0.97	0.98	0.06	0.08	--	--	--	--	--	--	--
Model 2	36.03	27	1.33	0.11	0.98	0.98	0.05	0.08	0.33	0.86	1	0.01	0.00	0.01	0.00
Model 3	41.48	31	1.33	0.09	0.98	0.98	0.05	0.08	5.78	0.80	5	0.01	0.00	0.01	0.00
Model 4	41.66	32	1.30	0.11	0.98	0.98	0.05	0.08	5.96	0.87	6	0.01	0.00	0.01	0.00

Note. Model 1 = without constraints; Model 2 = constrained covariances; Model 3 = constrained factor loadings; Model 4 = constrained covariances and factor loadings.

**Table 3:** Pearson correlations between WIFG and FIWG, antecedents, and outcomes variables.

WFG	LS	WH	RpS	FaS	FriS	Brood.	Refl.	Stand.	Disc.	Ext.	ES	Agr.	Conc.	Op.
WIFG	−0.12	0.24 *	0.01	−0.11	−0.01	0.24 *	0.07	0.02	0.12	0.01	−0.07	−0.04	0.00	−0.17 *
FIWG	0.03	0.19 *	0.01	−0.09	−0.04	0.06	0.09	0.13	0.29 *	−0.05	0.08	−0.09	−0.12	−0.04

Note. LS = life satisfaction; WH = worked hours; RpS = relevant person support; FaS = family support; FriS = friends support; Brood. = brooding; Ref. = reflection; Stand. = standards; Disc. = discrepancy; Ext = extraversion; ES = emotional stability; Agr. = agreeableness; Conc. = conscientiousness; OP = openness. * = *p* ≤ 0.05.

**Table 4:** Regression models to predict WIFG and FIWG.

	Women	Men
WIFG	*R*^2^ = 22.1% Worked hours (β = 0.39 ***) Brooding (β = 0.28 **)	*R*^2^ = 17.5% Brooding (β = 0.29 **) Worked hours (β = 0.23 *) Opennes (β = −0.22 *)
FIWG	*R*^2^ = 7.7% Discrepancy (β = 0.29 **)	*R*^2^ = 8.7% Discrepancy (β = 0.31 **)

Note. * = *p* ≤ 0.05; ** = *p* ≤ 0.01; *** = *p* ≤ 0.0.

## Data Availability

Data can be made available for consultation upon request to the corresponding author and with permission of the participants of the study.
